# Hormone Replacement Therapy advertising: sense and nonsense on the web pages of the best-selling pharmaceuticals in Spain

**DOI:** 10.1186/1471-2458-10-134

**Published:** 2010-03-16

**Authors:** Elisa Chilet-Rosell, Marta Martín-Llaguno, María Teresa Ruiz-Cantero, Pablo Alonso-Coello

**Affiliations:** 1 Preventive Medicine and Public Health Department, Universidad de Alicante, Alicante, Spain; 2 CIBER of Epidemiology and Public Health (CIBERESP), Spain; 3 Department of Audiovisual Communication and Publicity Department, Universidad de Alicante, Alicante, Spain; 4 Iberoamerican Cochrane Centre, Clinical Epidemiology and Public Health Department, Hospital Sant Pau, Barcelona, Spain

## Abstract

**Background:**

The balance of the benefits and risks of long term use of hormone replacement therapy (HRT) have been a matter of debate for decades. In Europe, HRT requires medical prescription and its advertising is only permitted when aimed at health professionals (direct to consumer advertising is allowed in some non European countries). The objective of this study is to analyse the appropriateness and quality of Internet advertising about HRT in Spain.

**Methods:**

A search was carried out on the Internet (January 2009) using the eight best-selling HRT drugs in Spain. The brand name of each drug was entered into Google's search engine. The web sites appearing on the first page of results and the corresponding companies were analysed using the European Code of Good Practice as the reference point.

**Results:**

Five corporate web pages: none of them included bibliographic references or measures to ensure that the advertising was only accessible by health professionals. Regarding non-corporate web pages (n = 27): 41% did not include the company name or address, 44% made no distinction between patient and health professional information, 7% contained bibliographic references, 26% provided unspecific information for the use of HRT for osteoporosis and 19% included menstrual cycle regulation or boosting feminity as an indication. Two online pharmacies sold HRT drugs which could be bought online in Spain, did not include the name or contact details of the registered company, nor did they stipulate the need for a medical prescription or differentiate between patient and health professional information.

**Conclusions:**

Even though pharmaceutical companies have committed themselves to compliance with codes of good practice, deficiencies were observed regarding the identification, information and promotion of HRT medications on their web pages. Unaffected by legislation, non-corporate web pages are an ideal place for indirect HRT advertising, but they often contain misleading information. HRT can be bought online from Spain, without a medical consultation or prescription constituting a serious issue for public health. In our information society, it is the right and obligation of public health bodies to ensure that such information is not misleading.

## Background

The balance between the benefits and risks of long term use of hormone replacement therapy (HRT) has been a matter of debate for decades. As early as 1939 the review published by Nancy Krieger in 2005 on the history and epidemiology of hormone replacement therapy (HRT) highlighted a potential association between HRT and breast cancer [1]. It was not until the 70s that the concern about endometrial cancer started [1]. Since the publication of the Women's Health Initiative (WHI) in 2002, detailing the higher incidence of cancer and cardiovascular disorders in women on HRT, the benefits of its long term use have been strongly questioned [2]. New clinical trials have confirmed these findings, concluding that the risk-benefit profile for the prevention of chronic conditions is generally unfavourable [3,4]. HRT is no longer recommended routinely as a preventative measure or as a first line treatment in the case of osteoporosis, nor for the prevention of chronic conditions [5] (only some institutions still challenge this line of though) [6]. Likewise, it is no longer indicated for asymptomatic menopausal women [5,7]. Consequently, there has been a steep drop in sales, documented mainly in Anglo-Saxon countries [1].

Since 2004, the Spanish Agency for Medicine and Health Care Products, in consonance with the regulatory agencies in other countries, leading health care institutions and most scientific societies, has considered HRT to be potentially indicated only in the treatment of severe climacteric symptoms with a negative effect on quality of life. In this case, they concur that the minimum effective dose should be used for the shortest possible duration [7,8].

HRT is classified as a prescription-only drug in Europe. That is, a medical prescription is required, and therefore advertising is only permitted where it is aimed at professionals authorised to prescribe, and not at the general public [9]. Nevertheless in some non European countries HRT direct to consumer advertising is allowed. Furthermore, online sales are illegal in the European Union (EU). Despite this fact, online sales of drugs in Europe have risen steeply since the 1990s, becoming not only a risk to public health but also a significant source of legal conflict between countries [10].

The EU has indicted the need to review the rigour, integrity and consistency of health information published on the Internet [11]. Spain adheres to the recommendations given in the European Code of Good Practice in the Promotion of Medicines adopted by the European Federation of Pharmaceutical Industries and Associations (EFPIA) [12]. This Federation requires each affiliated national association to constitute a Committee in order to ensure that companies fulfil the precepts of the Code, and to undertake disciplinary proceedings when appropriate. Specifically, Farmaindustria's Code of Practice Surveillance Unit in Spain supervises the Association's code and advises members [13]. Meanwhile, in Spain, it is the responsibility of the Jury of the Association for Self-Regulation of Commercial Communications to settle disputes and resolve disciplinary proceedings concerning the code.

The aim of the European Code of Good Practice in the Promotion of Medicines is to ensure that all pharmaceutical promotion methods, including the Internet, comply with current legislation [13]. Other objectives include ensuring that the ethical principles of professionalism and responsibility are upheld, that access to information aimed at professionals is restricted to the target audience and that the identity of the advertiser is clearly stated in the case of sponsored activities or formats. Furthermore, it supports the obligatory inclusion of information, comprising the Summary of Product Characteristics and scientific information based on valid data with scrupulous respect for truth and impartiality, all within the framework of a balanced, clear explanation. The need to avoid ambiguity is stressed, as is the need to base advertising claims on firm scientific evidence and medical opinion, which the reader is able to check up on (i.e. compliance with the code requiring inclusion of bibliographic references). Likewise, exaggeration or omission of data is forbidden [13].

In order to analyse the phenomena of HRT internet advertising in Spain, this research has three main objectives:

1. To estimate compliance with these regulations on the corporate web pages of the best-selling HRT drugs in Spain, in order to identify the degree of commitment of the corresponding pharmaceutical companies.

2. To analyse references to the same drugs on non-corporate web pages, establishing the name and address of the registered company and possible sponsors, in order to identify potential connections or ties with pharmaceutical companies.

3. To evaluate and quantify whether these prescription-only drugs are being sold on the Internet to the general public via online pharmacies based in Spain or abroad.

## Methods

In order to obtain the list of the best-selling and most frequently prescribed HRT brands in Spain, the Spanish Ministry of Health and Consumer Affairs General Directorate was consulted. These top eight products were Absorlent Plus, Activelle, Duofemme, Estalis, Estracomb, Merigest, Perifem and Progyluton.

Using the drugs' commercial brands, a search was carried out in January 2009, simulating the kind of search strategy that a typical internet user interested in locating information about these drugs would employ, using the Google search engine (the most popular search engine amongst internet users) [14]. All the web sites appearing on the first results page for each of the drugs were selected. In addition, when the first results page for each drug did not include the corporate web page, that is, the web site of the company marketing the drug in Spain, a further search was made in order to locate it, and the information about the drug analysed.

The corporate web sites were analysed for compliance with the following European Code's recommendations:

1. Restriction of the information to a specialised public (implemented through obligatory identification on behalf of the user in order to access the information). Specifically, the obligation to register - supplying the professional body membership number - in order to access information aimed at health care professionals, was monitored.

2. Obligatory inclusion of the drug's Summary of Product Characteristics.

3. Conformity of the indications with the Patient Information Leaflet (for this, the Patient Information Leaflet approved by the Spanish Agency of Medicine and Health Care Products for each drug was consulted).

4. Inclusion of the registered company name and address.

5. Inclusion of complete bibliographic references, including references to the original source.

Additionally, compliance with these same recommendations on non-corporate web pages referring to the same drugs was studied. If this information was not already explicitly stated on the page, the registered company name and address, and possible sponsorship, was identified using the website http://www.whois.net (this website offers domain-based research services).

The following information was also collected on non-corporate web pages:

- Whether the page offered links to sites selling medicines.

- Erroneous or inadequate information concerning drug indications.

The legal status of the online pharmacies was determined on the basis of the inclusion of the registered company name and address. In these cases, the availability of the drug's Summary of Product Characteristics, the stipulation of the need for a prescription and the presence of restrictions on access to the information, as well as the possibility of purchasing prescription-only medication from Spain was studied.

## Results

**Figure 1:**
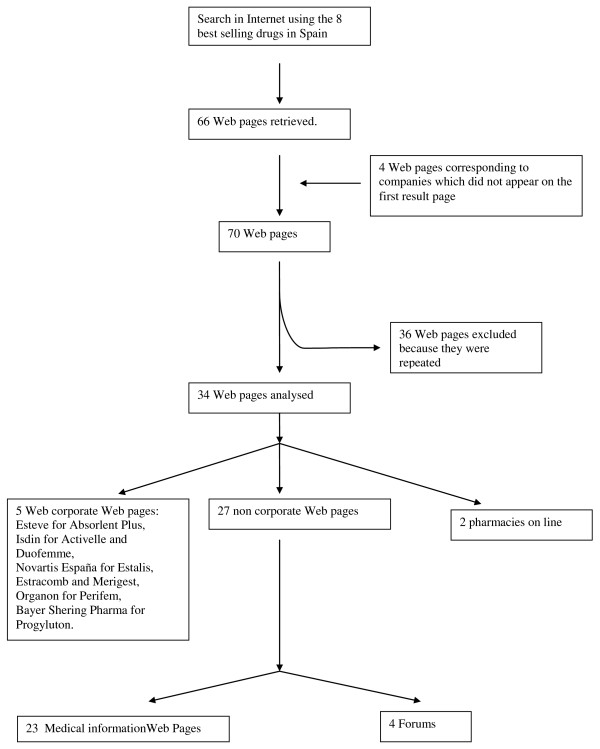
**Selection of Web of best selling HRT drugs in Spain to be analysed**.

Out of a total of 70 web pages (see Additional File 1), corresponding to the eight HRT drugs studied were retrieved from Google. After the exclusion of the repeated web pages, 34 were analysed (Figure 1). Of these 34 web pages, five were corporate web sites (Table 1), 27 were non-corporate (23 web pages providing medical information and four forums) (Table 2), and two were online pharmacies (Table 3).

**Table 1:** Compliance with Farmaindustria's "Code of Good Practice for the Promotion and Use of Medicines" on the web pages of pharmaceutical companies marketing the best-selling HRT drugs in Spain.

Web Site	Registration Required	Registered Company Name and Address Given	Summary of Product Characteristics	Therapeutic Indications	Bibliographic References
http://www.esteve.es	Must register, but professional body membership number not required	Yes	Yes	Yes	No references
http://www.isdin.es	Must register, but professional body membership number not required	Yes	Yes	Yes	No references
http://www.novartis.es	Must register, but professional body membership number not required	Yes	Information about HRT products does not appear	Information about HRT products does not appear	Information about HRT products does not appear
http://www.organon.es	Must register and provide professional body membership number	Yes	Information about Perifem does not appear	Information about Perifem does not appear	Information about Perifem does not appear
http://www.bayerscheringpharma.es	Registration not required	Yes	No (patient information is given)	Yes	No references

(Web pages consulted January 2009)

**Table 2:** Compliance with Farmaindustria's "Code of Good Practice for the Promotion and Use of Medicines" on non-corporate web pages (Information and Forums) promoting the best-selling HRT drugs in Spain.

Portal/HRT drug	Registered name/Co address	Data Sheet	Indications	Erroneous Indications	References	Differentiated patient physician information
http://www.dkvseguros.com (*): Absorlent Plus	Yes	No	No		No	No. Must consult a doctor
http://www.nomenclator.org (*): Absorlent Plus, Activelle, Duofemme, Estalis, Merigest, Perifem	No	No	No		No	No
http://www.vademecum.es (*): Absorlent Plus, Activelle, Duofemme, Estalis, Estracomb, Merigest, Perifem, Progyluton	Yes	No	Yes	Osteoporosis unspecific prevention	No	Yes, but does not require professional body membership number
http://www.epgonline.org (*): Absorlent Plus	Yes	No	Yes	Osteoporosis unspecific prevention	No	No
http://www.farmacopedia.com (*): Absorlent Plus, Activelle	No	No	No		No	No. Must consult a professional
http://www.diagnosticomedico.es (*): Absorlent Plus, Activelle, Estalis Merigest, Perifem	No	No	No		No	No
http://www.prospectos.net (*): Activelle	No	No	Yes		Yes	Yes, but does not require professional body membership number
http://www.agemed.es (*): Activelle, Duofemme, Merigest, Perifem, Progyluton	Yes	Yes	Yes		No	Yes, but does not require professional body membership number
http://www.laboratoriosilesia.com (*): Activelle	Yes	No	Yes		No	No
http://www.novasalud.cl (*): Activelle	Yes	No	Yes		Yes	Yes, but does not require professional body membership number
http://www.solucionestraumatologicas.com (*): Activelle	Yes	No	Yes		No	No
http://www.concursossanitarios.com/(*): Duofemme, Merigest, Perifem	Yes	No	No		No	No
http://www.famguerra.com (*): Estalis, Estracomb	No	Yes	Yes	Osteoporosis unspecific prevention	No	No
http://www.medbroadcast.com (*): Estalis, Estracomb	Yes	No	Yes		No	No. Must consult a professional
http://www.novartis.ca (*): Estalis, Estracomb	Yes	Yes	Yes		No	Yes, but does not require professional body membership number
http://www.facmed.unam.mx (*): Estalis, Estracomb	Yes	No	Yes	Osteoporosis unspecific prevention	No	No
http://www.saludzac.gob.mx (*): Estalis	Yes	No	Yes	Osteoporosis unspecific prevention	No	No
http://www.rxmed.com (*): Estracomb	No	Yes	Yes	Osteoporosis unspecific prevention	No	No. Must consult a professional
http://www.pdamecum.com (*): Estracomb	Yes	Yes	Yes		No	Yes, but does not require professional body membership number
http://www.healthyontario.com (*): Estracomb	Yes	No	Yes		No	No
http://chealth.canoe.ca/(*): Perifem	Yes	No	Yes		No	No. Must consult a professional
http://www.hipocrates.com (*): Progyluton	No	No	Yes		No	No
http://www.cmp-sanmartin.org (*): Duofemme, Perifem, Progyluton	Yes	No	Yes	Menstrual cycle Regulation/osteoporosis Prevention	No	No
http://www.enfemenino.com (**): Perifem, Progyluton	No.	No	Yes	Menstrual cycle Regulation	No	No
http://www.carlaantonelli.com (**): Progyluton	No	No	Yes	Boosts femininity/Menstrual cycle Regulation	No	No
http://www.portalesmedicos.com (**): Progyluton	No	No	Yes	Menstrual cycle Regulation	No	No
http://es.answers.yahoo.com (**): Progyluton	No	No	Yes	Menstrual cycle Regulation	No	No

(Web pages visited January 2009)

Type of web page: (*) Medical information, (**) Forum. Co.: Company

**Table 3:** Characteristics of Pharmacies on line promoting the best-selling HRT drugs in Spain.

Portal and HRT drug	Registered name/Co. address	Address of company http://www.whois.net	Data Sheet	Requirement	Differentiated patient/professional information	Sales in Spain
http://www.goldpharma.com: Absorlent plus, Duofemme, Estalis Estracomb, Merigest Perifem	No	Seychelles Islands	No	No	No	Burden of proof on the user to check current legislation for each country and accepted no responsibility
						
http://www.rx-med.net: Duofemme, Merigest Perifem	No	United Kingdom	No	No	No	Sells worldwide, but accepted no responsibility for compliance with legislation in other countries

(Web pages visited January 2009).

Table 1 includes the characteristics of the web pages provided by pharmaceutical companies marketing the HRT drugs analysed. Inclusion of the registered company name and address was the only issue fulfilled by them all. The application of measures to ensure that medication advertising was restricted to health professionals was apparent only in the case of one company (Organon Española SA.). Three other web pages required the user to register, but did not require a professional body membership number, whilst Bayer applied no control over access to information. Only two web pages complied with the precept of including the Summary of Product Characteristics, and three others presented the medication indications as given in the Spanish Drug Formulary. None of them included bibliographic references or cross-references to the original studies.

Table 2 presents the characteristics of non-corporate web pages offering medical information or forums where some of the medications studied were mentioned. Of the 27 non-corporate web pages, 11 (41%) provided no information concerning the registered company name and address. As for the obligation to include the Summary of Product Characteristics, 22 (81.5%) did not comply and a further five (18.5%) did not include the indications as provided in their corresponding Patient Information Leaflet.

Several inappropriate HRT therapeutic indications were recorded for 11 web pages: seven (26%) indicated HRT treatment for osteoporosis without mentioning a note of caution or the restrictions imposed by the Ministry for Health and Consumer Affairs due to the unfavourable risk-benefit profile [6,7]. Five (19%) indicated HRT as a means of regulating the menstrual cycle (four of these pages were forums), and one (4%) forum commented on the use of two HRT medications for "boosting femininity".

Two web pages (7%) providing medical information fulfilled the requirement of including bibliographic references and references to the original source. Results for providing different levels of information to patients and health professionals were mixed: 16 web pages made no distinction, five pages made no distinction but did insist on the need to consult a professional, five differentiated between patients and health professionals but did not restrict access to the information, and only one required the user to register in order to access the information, but did not require the user to prove their health professional status. Among the non-corporate web pages providing medical information, three included links to the sale of medications, although these did not include HRT drugs.

None of the two online pharmacies (http://www.goldpharma.com and http://www.rx-med.net) included had its base in Spain, although one was based in the European Union, specifically, the United Kingdom. None of them complied with the requirements analysed (inclusion of registered company name and address, Summary of Product Characteristics, prescription only status and differentiation between information aimed at the patient and that aimed at the health professional). In the case of these online pharmacies, medications could be purchased from Spain. Regarding legal status, Goldpharma (registered in the Seychelles, according to http://www.whois.net) placed the *onus probandi *(burden of proof) on the user to check current legislation for each country and accepted no responsibility. Likewise, Rx-med (registered in the UK, according to http://www.whois.net) sells worldwide, but accepted no responsibility for compliance with legislation in other countries.

## Discussion

Despite important efforts by the European Union to regulate and control medicines, our study shows that the European Code of Good Practice for the Promotion of Medicines has not had the desired effect on the quality of the information available on the best selling pharmaceuticals in Spain.

The corporate web pages of pharmaceutical companies owning HRT drugs do not comply with all the precepts laid down in this Code. On the other hand, forums and non-corporate web pages are promoting HRT irrespective of EU regulations. These websites contain misleading or erroneous information about the potential therapeutic uses of HRT, the information they provide is rarely based on scientific evidence, and they sometimes publicise online pharmacies. Similarly, the study confirmed a well-known fact that, despite EU prohibitions, it is still possible and easy to buy HRT medications from online pharmacies from anywhere in the world.

For women seeking information about the benefits and risks associated with HRT, there is a wealth of information available, but this is frequently contradictory. Much of this information is found in the media. For example, the first item retrieved by our search on the web page of the Spanish Agency for Medicines was the 2002 warning [15], where the use of HRT in the prevention of osteoporosis continued to be recommended. However, the 2004 [16] warning, restricting its use, did not appear during this search, whilst the 2008 warning appeared in second place, despite being the most current [17].

HRT has for decades been the source of a conflictive debate between pharmaceutical companies, epidemiologists, regulatory agencies and feminist groups. If a woman searches for HRT information online, the information she will find will be confusing, incomplete and frequently erroneous. In fact, women can even buy HRT online, without a prescription or medical advice and monitoring. This fact is particularly alarming since HRT drugs are powerful pharmacological agents shown to be thrombogenic and carcinogenic in the long term [2-4].

Significant inappropriate HRT indications were observed in the advertising published on the web pages analysed, for example indicating the use of HRT to prevent osteoporosis whilst failing to mention the restrictions imposed on this particular use [7,8]. Of greater concern is the fact that these sites promote other therapeutic uses for which the medication has not been approved, such as "boosting femininity" or regulating the menstrual cycle. These indications could involve a long-term use, which might constitute a significant health risk to the consumer, according to the evidence currently available [2-4].

Controlling the promotion and sale of medicines through indirect online marketing presents a challenge to European regulatory bodies. Unaffected by any regulations in this respect, forums, chat rooms and non-corporate web pages all constitute an ideal place to promote medications indirectly, and to broaden the scope of drugs' therapeutic applications. Thus, faced with the impossibility of generating direct demand for prescription-only drugs in Europe, pharmaceutical marketing may exploit these "disease mongering" strategies for menopausal and postmenopausal women [19-21]. Osteoporosis has been classified as a disease only in so far as it increases the risk of suffering a fracture [19,20], whilst the menopause, which is a normal part of the life cycle, is often advertised as pathological[21]. In both cases, HRT has been recommended and prescribed, leading to the perception of real diseases in the population [22].

At present, no specific body exists in Spain with the express remit of monitoring online sales of unauthorised medications. Due to the protection offered by the supranational structure of the Internet, countries possessing strict pharmaceutical controls have recently been overwhelmed with medications acquired in other countries with more permissive health legislations, and to date have been unable to prevent the phenomenon [9]. HRT drugs are sold in Spain on commercial web sites which exempt themselves from any responsibility regarding the purchase. Their registered headquarters are located in countries where such sales are either not legislated for, as is the case of sales from the Seychelles identified in this study, or illegal, as is the case of sales from the UK. Such uncontrolled sale of HRT drugs without a medical consultation or prescription constitutes a serious threat to public health, in this case more precisely to the women's population.

Since the pharmaceutical companies have a vested interest in the quality and ethical and legal status of the information provided about their products, it was the industry itself which agreed to implement self-regulation regarding promotion. Thus, the pharmaceutical companies committed themselves to compliance with codes of good practices for the promotion of medicines [13,14]. However, it may be that this strategic decision is actually one of the reasons behind the deficiencies observed regarding the identification, information and promotion of HRT medications on the Internet. Farmaindustria's Code of Practice Surveillance Unit has a great deal of work ahead of it if the corporate web pages are to be compelled to respect the codes of conduct. As this study has shown, in general, the companies do not require the user to register as a health care professional in order to access the information provided on the page, nor do the pages facilitate bibliographic references which provide objective support for the information. Furthermore, these companies do not include the necessary information concerning the pharmaceutical characteristics of their HRT drugs on their web pages.

Health information sites have proliferated, and in Spain there are now over 6,000 disease-related virtual forums [16]. These websites represent an important source of health information. The infringement of ethical requirements on these non-corporate web sites constitutes a danger to Public Health, given that 80% of patients now use the Internet to find information about their disorders [17]. Therefore, independently of official bodies, pharmaceutical companies should take the initiative in monitoring the suitability of information provided about their drugs on forum sites, chat rooms and non-corporate web pages. Furthermore, they ought to display the same concern over the quantity of information available on the Internet about their products as they do over unfair publicity from their peers [18].

The limitations of our study are related to the dynamic nature of the Internet, where content may vary each time a page is updated. Furthermore, it is possible that a search carried out at any time other than January 2009, could produce other web sites on the first results page. In addition, the selection of web sites did not comprise all the information and advertising available for each HRT drug analysed, as only the web sites appearing on the first results page were selected for analysis. Nevertheless, evidence suggests that users rarely look beyond the first results page [22].

Beyond the interests of pharmaceutical companies for the quality advertising of their products, and particularly in the promotion and sale of these products on the Internet, it is, as Muir Gray states, the right and obligation of public health bodies in an information society to ensure that such information is not misleading in terms of health, disease and pharmaceutical therapies. To date, the health authorities have yet to make a serious move in this direction [23].

## Conclusions

Controlling the promotion and sale of medicines through indirect online marketing represents a challenge to European regulatory bodies. Even though pharmaceutical companies have committed themselves to compliance with codes of good practice for the promotion of medicines, deficiencies were observed regarding the identification, information and promotion of HRT medications on their web pages. Unaffected by legislation, non-corporate web pages are an ideal place for indirect HRT advertising often containing misleading information. HRT can be bought online from Spain, without a medical consultation or prescription constituting a serious public health issue.

## Competing interests

The authors declare that they have no competing interests.

## Authors' contributions

ECR conceived the study and participated in the design of the study, acquisition of data from the web pages, analysis of data and coordination and draft of the paper. MML participated in the design of the study, provided knowledge on the regulation of advertising and participated on the draft of manuscript. MTRC participated in the design of the study, acquisition of data from the web pages and helped to draft the manuscript. PAC participated in the design of the study and helped to draft the manuscript.

All authors read and approved the final manuscript.

## Pre-publication history

The pre-publication history for this paper can be accessed here:

http://www.biomedcentral.com/1471-2458/10/134/prepub

## Supplementary Material

Additional file 1 **Annex 1**. List of the web sites retrieved in the search of the best selling HRT pharmaceuticals in Spain. Click here for file
