# Supplementary material for: Hormone Replacement Therapy advertising: sense and nonsense on the web pages of the best-selling pharmaceuticals in Spain
Source: BMC Public Health. 2010 Mar 16;10:134. doi: 10.1186/1471-2458-10-134 (PMC2850342; doi:10.1186/1471-2458-10-134)
Supplement: Additional file 1 — Annex 1. List of the web sites retrieved in the search of the best selling HRT pharmaceuticals in Spain. [file 1471-2458-10-134-S1.DOC]

**Additional File I. List of the web sites retrieved in the search of the best selling HRT pharmaceuticals in Spain**

**Absorlent Plus**

1. DKV Seguros: [www.dkvseguros.com/awa/dkvfarm/fm.asp?c=679472](http://www.dkvseguros.com/awa/dkvfarm/fm.asp?c=679472)
2. Nomenclator: [www.nomenclator.org/medicamento/absorlent-plus-8-parches-transdermicos.html](http://www.nomenclator.org/medicamento/absorlent-plus-8-parches-transdermicos.html)
3. Vademecum:[www.vademecum.es/medicamento/clasificacion-terapeutica/V/754/4681/1/1/absorlent.html](http://www.vademecum.es/medicamento/clasificacion-terapeutica/V/754/4681/1/1/absorlent.html)
4. Goldpharma: [www.goldpharma.com/?show=search&search_srt=ABSORLENT&lang=SPANISH](http://www.goldpharma.com/?show=search&search_srt=ABSORLENT&lang=SPANISH)
5. Epgonline:<http://www.epgonline.org/viewdrug.cfm/letter/A/language/LG0004/drugId/DR003389/drugName/Absorlent>
6. Farmacopedia: [www.farmacopedia.com/medicamento/679472/absorlent-plus-8-parches-transdermicos/](http://www.farmacopedia.com/medicamento/679472/absorlent-plus-8-parches-transdermicos/)
7. Diagnóstico médico: [www.diagnosticomedico.es/medicamento/Absorlent_Plus_8_Parches_Transdermicos](http://www.diagnosticomedico.es/medicamento/Absorlent_Plus_8_Parches_Transdermicos)

**Activelle**

1. Prospectos: [www.prospectos.net/activelle_comprimidos_recubiertos](http://www.prospectos.net/activelle_comprimidos_recubiertos)
2. Vademecum: [www.vademecum.es/medicamento/farmacologia/G/21758/1/activelle.html](http://www.vademecum.es/medicamento/farmacologia/G/21758/1/activelle.html)
3. Spanish Agency for Medicine and Health Care Products:

<https://sinaem4.agemed.es/consaem/especialidad.do?metodo=verFichaWordPdf&codigo=62496&formato=pdf&formulario=PROSPECTOS>

1. Nomenclator: [www.nomenclator.org/medicamento/activelle-28-comprimidos-recubiertos.html](http://www.nomenclator.org/medicamento/activelle-28-comprimidos-recubiertos.html)
2. Diagnóstico médico: [www.diagnosticomedico.es/medicamento/Activelle--47104.html](http://www.diagnosticomedico.es/medicamento/Activelle--47104.html)
3. Laboratorios Silesia: [www.laboratoriosilesia.com/silesia/productos/ficha.php?id=9](http://www.laboratoriosilesia.com/silesia/productos/ficha.php?id=9)
4. Novasalud: [www.novasalud.cl/vademecum/PRODUCTO/P5777.HTM](http://www.novasalud.cl/vademecum/PRODUCTO/P5777.HTM)
5. Soluciones traumatológicas: [www.solucionestraumatologicas.com/MFT/PRODUCTO/P5777.HTM](http://www.solucionestraumatologicas.com/MFT/PRODUCTO/P5777.HTM)
6. Farmacopedia: [www.farmacopedia.com/medicamento/753285/activelle-28-comprimidos-recubiertos/](http://www.farmacopedia.com/medicamento/753285/activelle-28-comprimidos-recubiertos/)

**Duofemme**

1. Vademecum: [www.vademecum.es/medicamento/farmacologia/G/26194/1/duofemme.html](http://www.vademecum.es/medicamento/farmacologia/G/26194/1/duofemme.html)
2. Spanish Agency for Medicine and Health Care Products: <https://sinaem4.agemed.es/consaem/especialidad.do?metodo=verFichaWordPdf&codigo=64718&formato=pdf&formulario=FICHAS>
3. Nomenclator: <http://nomenclator.org/medicamento/duofemme-28-comprimidos-recubiertos.html>
4. Goldpharma: www.goldpharma.com/?show=search&search_srt=DUOFEMME&lang=SPANISH
5. Enfemenino: www.foro.enfemenino.com/forum/.../__f21_f524-Alguien-toma-duofemme-para-menopausia-precoz.html
6. RX-med: [www.rx-med.net/fda/DUOFEMME.html](http://www.rx-med.net/fda/DUOFEMME.html)
7. Pdamecum: www.pdamecum.com/demo/Carpetas/D/Duofemme_/Duofemme_-dosis.html

**Estalis**

1. Vademecum: [www.vademecum.es/medicamento/farmacologia/G/22277/1/estalis.html](http://www.vademecum.es/medicamento/farmacologia/G/22277/1/estalis.html)
2. FamGuerra: [www.famguerra.com/Meds/Search/Meds.cfm?pagina=38646.htm](http://www.famguerra.com/Meds/Search/Meds.cfm?pagina=38646.htm)
3. Diágnostico médico: [www.diagnosticomedico.es/medicamento/Estalis--47106.html](http://www.diagnosticomedico.es/medicamento/Estalis--47106.html)
4. Medbroadcast: [www.medbroadcast.com/drug_info_details.asp?brand_name_id=1621](http://www.medbroadcast.com/drug_info_details.asp?brand_name_id=1621)
5. Novartis: [www.novartis.ca/products/en/estalis.shtml](http://www.novartis.ca/products/en/estalis.shtml)
6. Goldpharma: goldpharma.com/?lang=SPANISH&search_srt=ESTALIS&show=search
7. Facultad de Medicina UNAM: [www.facmed.unam.mx/bmnd/plm_2k8/src/prods/49684.htm](http://www.facmed.unam.mx/bmnd/plm_2k8/src/prods/49684.htm)
8. Nomenclator: nomenclator.org/medicamento/estalis-sequi-50-250-4-par-transd-fi-4-parch-fii.html
9. Servicios de Salud de Zacatecas: http://www.saludzac.gob.mx/plm/prods/38646.htm

**Estracomb**

1. Rxmed: http://www.rxmed.com/b.main/b2.pharmaceutical/b2.1.monographs/CPS-%20Monographs/CPS-%20(General%20Monographs-%20E)/ESTRACOMB.html
2. Medbroadcast: [www.medbroadcast.com/drug_info_details.asp?brand_name_id=1119](http://www.medbroadcast.com/drug_info_details.asp?brand_name_id=1119)
3. Facultad de Medicina UNAM: [www.facmed.unam.mx/bmnd/plm_2k8/src/prods/34008.htm](http://www.facmed.unam.mx/bmnd/plm_2k8/src/prods/34008.htm)
4. Vademecum: [www.vademecum.es/medicamento/farmacologia/G/4469/1/estracomb.html](http://www.vademecum.es/medicamento/farmacologia/G/4469/1/estracomb.html)
5. Fam Guerra: [www.famguerra.com/Meds/Search/Meds.cfm?pagina=34008.htm](http://www.famguerra.com/Meds/Search/Meds.cfm?pagina=34008.htm)
6. Novartis Canada: [www.novartis.ca/downloads/en/products/estracomb_patient_e.pdf](http://www.novartis.ca/downloads/en/products/estracomb_patient_e.pdf)
7. Healthy Ontario: [www.healthyontario.com/DrugDetails.aspx?brand_id=1119&brand_name=Estracomb](http://www.healthyontario.com/DrugDetails.aspx?brand_id=1119&brand_name=Estracomb)
8. Chealth: www.chealth.canoe.ca/drug_info_details.asp?brand_name_id=1119&rot=4
9. Goldpharma: www.goldpharma.com/?show=search&search_srt=ESTRACOMB&lang=SPANISH

**Merigest**

1. Vademecum: [www.vademecum.es/medicamento/farmacologia/G/21072/1/merigest.html](http://www.vademecum.es/medicamento/farmacologia/G/21072/1/merigest.html)
2. Goldpharma: www.goldpharma.com/?show=search&search_srt=MERIGEST&lang=SPANISH
3. Diagnostico: [www.diagnosticomedico.es/medicamento/Merigest_Sequi_2mg_28_Comprimidos_Recub--88611.html](http://www.diagnosticomedico.es/medicamento/Merigest_Sequi_2mg_28_Comprimidos_Recub--88611.html)
4. Concursos Sanitarios: [www.concursossanitarios.com/Producto-MERIGEST_2MG_28_COMPRIMIDOS-Lcicgcbgjggfe.xsql](http://www.concursossanitarios.com/Producto-MERIGEST_2MG_28_COMPRIMIDOS-Lcicgcbgjggfe.xsql)
5. Nomenclator: nomenclator.org/medicamento/merigest-sequi-2mg-28-comprimidos-recub.html
6. Spanish Agency for Medicine and Health Care Products: <https://sinaem4.agemed.es/consaem/especialidad.do?metodo=verFichaWordPdf&codigo=61774&formato=pdf&formulario=FICHAS>
7. Rx-med: [www.rx-med.net/fda/Merigest.html](http://www.rx-med.net/fda/Merigest.html)

**Perifem**

1. Vademecum: [www.vademecum.es/medicamento/farmacologia/G/5107/1/perifem.html](http://www.vademecum.es/medicamento/farmacologia/G/5107/1/perifem.html)
2. Hipocrates: [www.hipocrates.com/vademe/buscaprodu.phtml?quehacer=1&medica=PERIFEM](http://www.hipocrates.com/vademe/buscaprodu.phtml?quehacer=1&medica=PERIFEM)
3. En femenino: foro.enfemenino.com/forum/f91/__f20960_f91-Alguien-ha-tomado-alguna-vez-perifem.html
4. Spanish Agency for Medicine and Health Care Products: [www.sinaem4.agemed.es/consaem/especialidad.do?metodo=verFichaWordPdf&codigo=60232&formato=pdf&formulario=PROSPECTOS](https://sinaem4.agemed.es/consaem/especialidad.do?metodo=verFichaWordPdf&codigo=60232&formato=pdf&formulario=PROSPECTOS)
5. Nomenclator: www.nomenclator.org/medicamento/perifem-21-comprimidos.html
6. My forum: www.my-forum.org/descripcion.php?numero=462&nforo=106648&pag=130
7. Concursos Sanitarios: [www.concursossanitarios.com/Producto-PERIFEM_21_COMPRIMIDOS-Lcjacaaiaeahb.xsql](http://www.concursossanitarios.com/Producto-PERIFEM_21_COMPRIMIDOS-Lcjacaaiaeahb.xsql)
8. Rx-Med: [www.rx-med.net/fda/PERIFEM.html](http://www.rx-med.net/fda/PERIFEM.html)
9. Diagnostico Médico: [www.diagnosticomedico.es/medicamento/Perifem_21_Comprimidos--90368.html](http://www.diagnosticomedico.es/medicamento/Perifem_21_Comprimidos--90368.html)

**Progyluton**

1. Bayerscheringpharma: [www.bayerscheringpharma.es/ebbsc/cms/es/_galleries/download/s_mujer/prospectos/Progyluton.pdf](http://www.bayerscheringpharma.es/ebbsc/cms/es/_galleries/download/s_mujer/prospectos/Progyluton.pdf)
2. En femenino: [www.foro.enfemenino.com/forum/matern4/__f41551_matern4-Alguien-toma-progyluton-o-conoce-este-medicamento.html](http://www.foro.enfemenino.com/forum/matern4/__f41551_matern4-Alguien-toma-progyluton-o-conoce-este-medicamento.html)
3. [Portales médicos: www.portalesmedicos.com/foros_medicina_salud_enfermeria/ubbthreads.php/posts/43439/Progyluton](http://www.portalesmedicos.com/foros_medicina_salud_enfermeria/ubbthreads.php/posts/43439/Progyluton)
4. Vademecum: www.vademecum.es/medicamento/farmacologia/G/3287/1/progyluton.html
5. [Yahoo. www.es.answers.yahoo.com/question/index?qid=20070721095400AAqHMIu](http://es.answers.yahoo.com/question/index?qid=20070721095400AAqHMIu)
6. [CMP San Martinwww.cmp-sanmartin.org/plm/PLM/productos/31985.htm](http://www.cmp-sanmartin.org/plm/PLM/productos/31985.htm)
7. Carla Antonelli: [www.carlaantonelli.com/foros/viewtopic.php?f=29&t=9239&start=0&st=0&sk=t&sd=a](http://www.carlaantonelli.com/foros/viewtopic.php?f=29&t=9239&start=0&st=0&sk=t&sd=a)
8. Spanish Agency for Medicine and Health Care Products: [www.sinaem4.agemed.es/consaem/especialidad.do?metodo=verFichaWordPdf&codigo=53201&formato=pdf&formulario=FICHAS](http://www.sinaem4.agemed.es/consaem/especialidad.do?metodo=verFichaWordPdf&codigo=53201&formato=pdf&formulario=FICHAS)
9. Rx-Med: [www.rx-med.net/fda/PROGYLUTON.html](http://www.rx-med.net/fda/PROGYLUTON.html)
